# Osteoprotective Roles of Green Tea Catechins

**DOI:** 10.3390/antiox9111136

**Published:** 2020-11-16

**Authors:** Hsuan-Ti Huang, Tsung-Lin Cheng, Sung-Yen Lin, Cheng-Jung Ho, Joanna Y. Chyu, Rong-Sen Yang, Chung-Hwan Chen, Chwan-Li Shen

**Affiliations:** 1 Orthopaedic Research Center, Kaohsiung Medical University, Kaohsiung 80701, Taiwan; hthuang@kmu.edu.tw (H.-T.H.); junglecc@gmail.com (T.-L.C.); tony8501031@gmail.com (S.-Y.L.); rick_free@mail2000.com.tw (C.-J.H.); 2 Department of Orthopedics, Kaohsiung Medical University Hospital, Kaohsiung Medical University, Kaohsiung 80701, Taiwan; 3 Departments of Orthopedics, College of Medicine, Kaohsiung Medical University, Kaohsiung 80701, Taiwan; 4 Department of Orthopedics, Kaohsiung Municipal Ta-Tung Hospital, Kaohsiung 80145, Taiwan; 5 Regeneration Medicine and Cell Therapy Research Center, Kaohsiung Medical University, Kaohsiung 80701, Taiwan; 6 Department of Physiology, College of Medicine, Kaohsiung Medical University, Kaohsiung 80701, Taiwan; 7 School of Medicine, University of Texas Medical Branch, Galveston, TX 77555, USA; jychyu@utmb.edu; 8 Department of Orthopedics, National Taiwan University Hospital, Taipei 100229, Taiwan; rsyang@ntuh.gov.tw; 9 Institute of Medical Science and Technology, National Sun Yat-Sen University, Kaohsiung 80424, Taiwan; 10 Department of Healthcare Administration and Medical Informatics, Kaohsiung Medical University, Kaohsiung 80701, Taiwan; 11 Department of Pathology, Texas Tech University Health Sciences Center, Lubbock, TX 79430, USA; 12 Center of Excellence for Integrative Health, Texas Tech University Health Sciences Center, Lubbock, TX 79430, USA

**Keywords:** green tea extract, osteoprotection, apoptosis, antioxidant, inflammation, mesenchymal stem cells

## Abstract

Osteoporosis is the second most common disease only secondary to cardiovascular disease, with the risk of fracture increasing with age. Osteoporosis is caused by an imbalance between osteoblastogenesis and osteoclastogenesis processes. Osteoclastogenesis may be enhanced, osteoblastogenesis may be reduced, or both may be evident. Inflammation and high reactive oxygen enhance osteoclastogenesis while reducing osteoblastogenesis by inducing osteoblast apoptosis and suppressing osteoblastic proliferation and differentiation. Catechins, the main polyphenols found in green tea with potent anti-oxidant and anti-inflammatory properties, can counteract the deleterious effects of the imbalance of osteoblastogenesis and osteoclastogenesis caused by osteoporosis. Green tea catechins can attenuate osteoclastogenesis by enhancing apoptosis of osteoclasts, hampering osteoclastogenesis, and prohibiting bone resorption in vitro. Catechin effects can be directly exerted on pre-osteoclasts/osteoclasts or indirectly exerted via the modulation of mesenchymal stem cells (MSCs)/stromal cell regulation of pre-osteoclasts through activation of the nuclear factor kB (RANK)/RANK ligand (RANKL)/osteoprotegerin (OPG) system. Catechins also can enhance osteoblastogenesis by enhancing osteogenic differentiation of MSCs and increasing osteoblastic survival, proliferation, differentiation, and mineralization. The in vitro effects of catechins on osteogenesis have been confirmed in several animal models, as well as in epidemiological observational studies on human subjects. Even though randomized control trials have not shown that catechins provide anti-fracture efficacy, safety data in the trials are promising. A large-scale, placebo-controlled, long-term randomized trial with a tea regimen intervention of optimal duration is required to determine anti-fracture efficacy.

## 1. Osteoporosis

Osteoporosis is a disease that causes reduced bone density and quality. Bones become fragile and more porous, and as a result the risk of fracture is greatly increased. As a result, osteoporosis is a global problem with increasing laboratory significance. Bone mineral density (BMD) and quality decide bone strength. Bone quality is determined by bone architecture, turnover, damage accumulation, matrix mineralization, and collagen composition [1]. The lifetime risk of fragility fractures is around 40%, including forearm, vertebral, and hip fractures, similar to the risk for cardiovascular disease [2]. One in two women and one in three men aged greater than 60 years will have an osteoporosis-related fragility fracture that may increase the monetary burden by USD131.5 billion by 2050 [3,4,5].

Bone has dynamic turnover through a remodeling process controlled by osteoclasts, osteoblasts, and osteocytes. Mesenchymal stem cells (MSCs) in bone marrow can differentiate into osteoblastic linage cells containing osteoblasts and osteocytes [6], whereas osteoclasts are derived from the monocyte/macrophage linage of hematopoietic stem cells [7]. The metabolic activities of these cells are regulated by local and systemic stimuli comprising mechanical, immunological, and hormonal factors. An imbalance of regulatory factors is closely associated with osteoporosis. An imbalance between osteoclastogenesis (bone resorption) and osteoblastogenesis (bone formation) during remodeling, which causes enhanced bone resorption with or without decline in bone formation, remains the main cause of osteoporosis [8]. 

### 1.1. Molecular Regulation in Osteoporosis

The canonical Wnt signaling is the main pathway regulating osteoblastogenesis, including osteoblastic differentiation, proliferation, maturation, and activity. Wnt signaling is associated with an increase of intracellular and intranuclear translocation of β-catenin levels [9]. Both runt-related transcription factor 2 (Runx2) [10] and Osterix (Osx) [11], also known as SP7 in human [12], control osteoblastic differentiation of MSCs [9]. Osteogenic differentiation can be enhanced by bone morphogenetic proteins (BMPs), a member of the transforming tumor growth factor (TGF) superfamily, through increasing Runx2 expression, which in turn raises Osx/SP7 expression [13]. Conversely, Wnt signaling inhibits adipogenesis by stimulating Runx2 and inhibiting CCAAT-enhancer binding protein α (C/EBP α) [13]. Excessive oxidative stress may cause increased reactive oxygen species (ROS), increased phosphorylation of p53 and p66, and decreased expression of β-catenin, leading to apoptosis of osteoblasts [14,15,16,17]. In addition, the increase in osteoblast apoptosis is a possible mechanism for the reduced bone-forming ability of senescent bone [18].

The mononuclear cell precursors of the monocyte/macrophage lineage of hematopoietic stem cells differentiate into osteoclasts, which are large multinucleated cells. Osteoclastogenesis is mainly controlled by macrophage-colony stimulating factor (M-CSF) and by receptor activation of the nuclear factor kB (RANK)/RANK ligand (RANK-L)/osteoprotegerin (OPG) system [7]. Mature multinucleated osteoclasts lead to bone resorption and initiation of bone remodeling [7]. Age-dependent enhancement in osteoblast-regulated osteoclastogenesis has shown decreased OPG expression and augmented expression of RANKL and M-CSF in stromal/osteoblastic cells of the elderly [19,20]. Inflammatory cytokines, including IL-1, TNFα, IL-6, and IL-17, enhance osteoclastogenesis and prohibit the differentiation and function of osteoblasts. Moreover, interferon (IFN)-γ, IL-10, IL-4, and IL-12 prohibit osteoclastogenesis, while IL-4 enhances osteoblast migration and proliferation and inhibits osteoblast differentiation [13].

### 1.2. Oxidative Stress is Related to Osteoporosis 

ROS surge is one of the leading causes of osteoporosis during bone aging. An increase in oxidative stress or chronic inflammation, as shown by excessive reactive oxygen species (ROS), usually causes injury to DNA, protein, and lipids and promotes the progression of osteoporosis [21,22,23,24]. Apoptosis of osteoblasts and osteocytes with decreased osteoblast count can be induced by oxidative stress due to the activation of nuclear transcription factor kB (NF-kB) and extracellular signal-regulated kinases (ERK) signaling pathways [25], which represses the bone formation rate via Wnt/β-catenin signaling pathways in osteoblastogenesis [26]. ROS is involved in bone resorption through direct bone degradation by osteoclast-generated superoxide or through enhancement of osteoclast differentiation and function [22,23,27,28,29,30,31].

Age-dependent bone loss is associated with reduced osteoblast numbers, enhanced apoptosis of osteoblasts and osteocytes, and decreased rate of bone formation [32,33,34]. Osteoporosis-related fragility fractures are noted in a murine model of premature aging and are characteristic of oxidative stress [35]. In estrogen-deficient females, elevated ROS and promoted phosphorylation of p66shc and p53 proteins that enhance production of mitochondrial ROS and cell apoptosis were found [14]. Previous human studies have also indicated a reverse relationship between the level of ROS and level of BMD. Since oxidative stress can cause bone loss, decreasing oxidative stress through antioxidants could be a possible strategy to decrease bone loss for osteoporosis prevention. Therefore, regulation of excessive oxidative stress could be a potential target for the management of osteoporosis. The bone healthy effects of antioxidant have also been shown in human studies [15,16]. Green tea catechins are potent antioxidants that have been extensively studied for their joint and muscle protective effects [36]. In this review, we examine the effects of green catechins on bone in the literature.

## 2. Catechins

Tea, brewed from the dried leaves of Camellia sinensis, is a common drink. In 2010, global consumption of tea was 4.52 million tons. Most of the teas are black tea and green tea [37]. Catechins (3,3′,4′,5,7- pentahydroxyflavan), which account for more than 80% of green tea polyphenols, are derived from flavan-3-ol. (−)-epicatechin (EC), (−)-epicatechin gallate (EGC), (−)-epigallocatechin (EGC) and (−)-epigallocatechin gallate (EGCG) are the main types of catechins [38]. EGCG possesses the most potent antioxidant and free radical scavenging abilities [39,40]. Thus, many benefits of green tea come from the free radical scavenging activity and antioxidant effects of catechins [36,41,42,43,44,45,46,47,48,49,50,51].

### 2.1. In Vitro Effect of Catechins

The antiresorptive properties of bioactive components in green tea can enhance apoptosis of osteoclasts, hamper osteoclastogenesis, and prohibit bone resorption in vitro. Effects can be directly exerted on pre-osteoclasts/osteoclasts or via the modulation of MSCs/stromal cell regulation of pre-osteoclasts. EGCG treatments have been shown to increase apoptosis of osteoclast-like multinucleated cells through a Fenton reaction, whereas osteoblasts were spared from apoptosis [52]. Yun et al. reported EGCG (20 μM)-decreased mRNA expression of MMP-9, but not MMP-13, and -2 in murine calvarial primary osteoblastic cells. Moreover, in the co-culture system, EGCG significantly prohibited osteoclast formation at this concentration. The authors indicated that EGCG may decrease alveolar bone resorption in periodontal diseases by prohibiting MMP-9 expression in osteoblasts and osteoclastogenesis [53]. Oka et al. found activities of MMP-9 and MMP-2 decline in EGCG (10 and 100 μM)-treated rat pre-osteoclast cell cultures, while EGCG prohibits osteoclastogenesis by MMPs inhibition. In addition, theaflavin-3,3’-digallate (TFDG), the polyphenol found in black tea, suppressed actin ring formation more significantly than EGCG [54]. Tokuda et al. reported EGCG decreases bone resorption by suppression of IL-6 secretion [55] or through suppressing the activation of p44/p42 MAP kinase [56] via osteoblastic linage cell regulation. EGCG represses osteoclastic differentiation via downregulation of the RANKL-induced expression of the nuclear transcription factor of activated T cells c1, subsequently inhibiting osteoclastogenesis and causing decreased bone resorption [57,58]. Lin et al. found EGCG, 10–100 μM, decreased the RANKL-induced osteoclastogenesis and pit formation in monocyte, RAW 264.7 cells, and primary bone marrow macrophage (BMM) cells via the inhibition of RANKL-induced NF-kB transcriptional activity and nuclear translocation [59]. Chen et al. compared the effects of EC, ECG, EGC, and EGCG at a concentration of 1 μM, the approximate concentration found in a cup of tea, to concentrations of 10 μM and 100 μM, in murine bone marrow pluripotent mesenchymal cells, D1. EGCG was shown to increase mRNA expression of OPG [60]. Chen et al. found that EGCG at low concentrations (1 and 10 µM) decreases RANKL/OPG ratio, and both mRNA expression and protein secretion then decrease osteoclastogenesis via tartrate resistant acid phosphatase (TRAP) (+) stain osteoclasts and TRAP activity by the RANK/RANKL/OPG pathway in co-culture of ST2 feeder cells and RAW 264.7 cells [61]. 

EGCG also regulate RANK/RANKL/OPG pathway in osteoblast, MC3T3-E1 [62] and primary osteoblastic cells isolated from newborn mouse calvariae [63] via prostaglandin E2 (PGE2). EGCG treatment decreased LPS-induced expression of mRNA expression of RANKL in primary osteoblasts [63] and increased mRNA expression of OPG in MC3T3-E1 [62]. Both effects were through PGE2.

The bioactive components in green tea show the beneficial effects on osteoblastogenesis via increased osteoblastic survival, proliferation, differentiation, and mineralization [37]. Chen et al. evaluated the effects of EGCG (1 and 10 μM) in D1 cells, a murine bone marrow mesenchymal cell line. EGCG at 1 and 10 μM increases mRNA expression of the degradation of Runx2, Osx/SP7, osteocalcin, and alkaline phosphatase (ALP). EGCG treatment also significantly enhanced the ALP activity. Furthermore, mineralization was augmented after EGCG treatment, with the higher concentration being more effective, while a 24-h EGCG treatment hampered the proliferation of D1 cells [64]. Thus, EGCG at 1 and 10 µM can enhance mRNA expression, including Runx2, BMP-2, ALP, osteocalcin, and osteonectin, ALP activity, and mineralization in human bone marrow MSCs. EGCG without antioxidant activity can also enhance mineralization of human bone marrow MSCs [65]. EGCG enhanced osteoblasts survival by decreasing the production of TNF-α and IL-6 [66]. EGCG, 1–5 μM, induced a dose-dependent enhancement of ALP activity and mineralization in SaOS-2 cells, which are human osteoblast-like cells. However, EGCG decreased protein levels of Runx2 at a late stage, which may indicate the facilitation of osteogenic differentiation at an early stage [67]. EGCG also enhances osteoblastogenesis via the Wnt/β-catenin signaling pathway [68].

In osteoblast-like MC3T3-E1 cells, endothelin-1 enhances production of a potent bone resorptive agent, IL-6 [69]. In MC3T3-E1 cells and primary cultured mouse osteoblasts, EGCG significantly decreases IL-6 secretion upon endothelin-1 treatment via inhibition of the p44/p42 MAP kinase pathway, and the suppressive effect is the crosstalk between Raf-1 and PKC in the endothelin-1 signaling pathway [55,70]. In osteoblasts, EGCG increased prostaglandin F2a-related vascular endothelial growth factor production by enhancing Jun N-terminal kinase, but does not affect phosphorylation of p38 MAP kinase or ERK1/2 [56,71].

MiRNAs are small non-coding endogenous RNA molecules (around 19–25 nucleotides in length), which regulate post-transcriptional gene expression [72,73,74,75,76]. Five miRNAs (miR-21-5p, miR-24-3p, miR-93-5p, miR-100-5p, and miR-125b-5p) were found also significantly upregulated in bone tissue from osteoporotic patients compared to controls [77]. MiR-30b-5p and miR-142-3p were significantly correlated with both total hip and femur neck BMD [78]. EGCG treatment prohibited the apoptosis in MSCs induced by hypoxia and increased the level of RUNX2, BMP-2, ALP, and PINP in MSCs via the upregulated miR-210 expression [79].

EGCG receptors have been studied for years. The 67-kDa laminin receptor (67LR) was found [80,81,82]. 37-kDa gene proteins are a monomeric forerunner to a 67-kDa dimmer [83]. Retinoic acid promotes the attaching of EGCG to cancer cells via enhancing the expression of 67LR [84]. Several previous studies found that the suppressive effects of EGCG on cancer cell growth were through the expression of 67LR [77,78,79,80,81]. Moreover, the anti-allergic and anti-inflammatory effects of EGCG were also via the 67LR [82,83,84]. After EGCG binding to 67LR, through eukaryotic translation elongation factor 1A (eEF1A), the phosphorylation of MYPT1 at Thr-696 is reduced, which stimulated myosin phosphatase, and the actin cytoskeleton is rearranged, which can inhibit cell growth. In multiple myeloma and acute myeloid leukemia, EGCG persuades apoptosis in the 67LR-expressing cells [85]. The effects of EGCG on 67LR may be the most important mechanisms for the bio-effects of EGCG (Table 1). 

### 2.2. In Vivo Effects of Catechins

The in vitro effects of catechins on osteogenesis have been confirmed in several bone-loss models in animal studies including estrogen deficiency [86,87], aging [87,88], testosterone deficiency [88], caloric restriction [89], obesity [90], glucocorticoid-induced [91], chronic inflammation [92], and others [93].

Shen et al. demonstrated that feeding water with green tea polyphenols (GTP) consisting of EGCG, ECG, EC, EGC, and catechins, at 0.1% and 0.5% (*w/v*, *w/v*) to 14-month-old F344xBFN1/NIA OVX and sham female rats for 16 weeks showed increased urinary ECG and EC concentrations in a dose-dependent and time dependent manner. GTP supplementation ameliorated bone loss on BMD examination and deterioration of microarchitecture on histomorphometric studies and eventually enhanced bone strength on mechanical tests [86,87]. Doses of 0.1% and 0.5% GTPs (about 1 and 4 cups of green tea of daily consumption) are safe without significant changes to serum total protein, albumin, creatinine, blood urea nitrogen, creatinine phosphokinase, aspartate transferase, alanine transferase calcium, phosphate, glucose, total bilirubin, alkaline phosphatase activity, globulin, amylase, or cholesterol [86]. Chen et al. also evaluated the in vivo effects of EGCG on estrogen deficiency by intra-peritoneal injection. They found that EGCG, 3.4 mg/kg/day (about 10 μM in the serum), attenuated the BMD drop and ameliorated parameters including the trabecular number, bone volume, trabecular thickness, and trabecular separation in μCT examination compared to non-treated ovariectomized rats. In the third lumbar spine, comparable improvements in trabecular thickness and bone volume were also noted. In addition, EGCG treatment increased the bone volume in the tibial cortex. Increased BMP-2 expression may be one of the effective mechanisms for EGCG. No significant systemic toxic effect after EGCG treatment was found in the serial biochemistry data [94]. In male rats, Shen et al. found that GTP supplementation (0.5% *w/v* in potable water) ameliorated trabecular and cortical bone loss. A dual effect of GTP was identified through enhanced bone formation and declined bone resorption via GTP’s antioxidant capacity [88]. Chen’s team evaluated the effect of EGCG in vivo in male rats via local injection at a low dose. Lin et al. found that percutaneous local injection of EGCG for 2 weeks, 10 µM/L, 40 µL, enhances callus bone volume and increases the tibial mechanical properties. The break load, maximal load, Young’s modulus, and stiffness improved after EGCG treatment with more BMP-2 expression than that found in male rats without EGCG treatment in a tibia fracture model [95]. Lin et al. also demonstrated that local EGCG (10 µM/L, 40 µL) improves femoral defect healing with de novo bone formation by enhancing the bone volume and improving the mechanical properties similar to the above study. With increased expression of BMP-2, EGCG enhanced de novo bone formation by improving max load, ultimate stress, max load, break point, break point, and stiffness [96].

EGCG has also been studied in glucocorticoid-induced osteoporosis (GIO). Xi et al. found EGCG injection (0.5 mg/kg/day) for 4 weeks improved the cancellous structure in proximal tibia metaphysis in GIO. EGCG reduced peroxisome proliferator-activated receptor γ protein expression and enhanced β-catenin, Wnt, and cyclin D1 as part of the mechanism [91]. Liu et al. found EGCG pretreatment significantly protected osteoblast viability from exposure to dexamethasone (DEX). In addition, ALP and SOD activities and mineralization were also improved. In osteoblasts, promoting the nuclear factor erythroid-derived 2-like-2 (Nrf2) pathway at both the cellular and mitochondrial levels may be one of the mechanisms in EGCG that reduces DEX-induced ROS. EGCG also reduced osteoblast apoptosis and increased 11β-hydroxysteroid dehydrogenase activity. In the femora, EGCG improved bone microstructure and ameliorated deterioration of bone quality induced by DEX [97].

Shen’s team also evaluated the effects of GTP in a systemic chronic inflammation lipopolysaccharide (LPS)-induced bone loss model of adult rats. GTP at 0.5% *w/v* for 12 weeks led to higher values of BMD, bone mineral content (BMC), and serum osteocalcin, but decreased serum TRAP. GTP was shown to increase bone strength by attenuating oxidative stress-induced damage and inflammation [98,99]. Moreover, there was a synergistic osteoprotective effect of GTP and alfacalcidol on bone properties as shown by an increased bone mass, restored LPS-induced deleterious effects in the femur, proximal tibia and endocortical tibial shaft, maintained bone microarchitecture and femoral strength, and decreased serum TRAP on LPS-induced bone loss model [100]. Such synergistic effects are mediated through the suppression of 8-hydroxy-2′-deoxyguanosine and tibia mRNA expression of tumor necrosis factor-α (TNF-α) induced by LPS in urine [100].

Shen et al. found GTP use decreased fat-free mass and ameliorated bone properties via improving bone microarchitecture, BMD, and strength in obese rats in a high-fat diet-induced bone deterioration model. Effects of GTP were brought forth through suppression of bone remodeling, leading to greater bone volume [101,102]. The bone-protective effects of GTP are similar in obese rats before and after switching to a caloric-restricted diet. Common effects of GTP included increased femoral trabecular thickness and number, mass and strength, and cortical thickness of tibia, as well as decreased insulin-like growth factor-I and leptin. EGCG also attenuated formation rate, trabecular separation, and eroded surface at proximal tibia [89]. Shen et al. demonstrated the beneficial effects of GTP on bone formation at both lumbar vertebrae and femur in high-fat diet and caloric-restricted diet models [89].

In the model of periodontal disease, the protective effects of GTP on alveolar bone have also been extensively studied. Nakamura et al. demonstrated oral green tea catechins attenuated LPS-induced alveolar bone loss in BALB/c mice by reducing IL-1β production or by directly decreasing osteoclastogenesis [103]. Yoshinaga et al. found that green tea extract decreased inflammatory cell infiltration and RANKL expression, eventually leading to loss of alveolar bone resorption in a rat model [104]. Cai et al. found 0.02% EGCG in drinking water reduced bone loss in a rat model of experimental periodontitis. EGCG can reduce inflammatory serum mediators by decreasing IL-1β, TNF-α, IL-17, IL-6, and other mediators, but not IL-23 [105].

Gennaro et al. found that green tea treatment decreased TNF-α expression in periodontal disease and ameliorated alveolar bone resorption in diabetic rats [106]. Jin et al. found EGCG prohibited titanium particle-induced osteolysis by inhibiting TNF-α expression and osteoclast formation [107]. Almeida et al. found that topical green tea extract solution (GTE) decreased inflammatory process including decreasing TNF-α and IL-1β while increasing IL-10 in experimental periodontitis. Moreover, fewer TRAP-positive multinucleated osteoclasts and higher bone in furcation were found with GTE treatment [46].

A high dose of GTE may actually harm bones by acting as a pro-oxidant. Iwaniec et al. found that green tea extract (1% and 2%, *w/w* in diet) for 6 weeks was harmful to bone growth in growing male mice, with reduced cortical bone volume and thickness, shorter bone length, and compromised BMC [108] (Table 2).

### 2.3. Human Studies of Catechins

#### 2.3.1. Epidemiological Observational Studies

The diagnostic criteria of osteoporosis were set by the standard deviation (SD) scores of BMD related to the peak bone mass in healthy young women. Osteoporosis is defined as a T score of −2.5 or less in BMD, and low bone mass (osteopenia) is stated as having a BMD T-score between −1 and −2.5 [110]. Hip fractures and vertebral fractures are prototypical osteoporotic fractures and intensely associated with reductions in hip and spine BMD [111]. Since BMD is the golden standard for osteoporosis diagnosis, following up on the effects of tea on BMD is required for evaluating the potential application of tea on osteoporosis prevention. Currently, overall bone strength cannot be measured precisely; thus, BMD is widely used as a proxy for bone strength, as BMD accounts for about 70% of bone strength. BMD is highly correlated with bone strength and is one of the best predictors for osteoporotic fracture [1,112]. In clinical practice, we use dual-energy X-ray absorptiometry (DXA) to quantify BMD at the central skeleton including the femoral neck, spine, and hip [113]. BMD can be used to predict fracture risk, monitor the disease progression or monitor response to osteoporosis medication two years after the inception of therapies [114,115,116,117,118]. Bone turnover markers (BTMs) can be effective tools to evaluate therapy short-term due to the limited availability, cost, and inconvenience of DXA and the delay in detecting BMD changes after treatment initiation. Serum C-terminal telopeptide of type 1 collagen (CTX) and procollagen type 1 N propeptide (P1NP) are good tools for short-term monitoring to evaluate the responses to osteoporosis treatment and can be the base for appropriately adjusting treatment regimens earlier than BMD [119], but not associated with hip fracture risk [120].

Several epidemiological observational studies reported the bone health promoting effects of tea. Habitual tea drinkers with higher BMD have been reported among post-menopausal women in the United Kingdom [121], Canada [122], and the United States [123], as well as among both men and women in Taiwan [124]. Other studies also verified the protection effect of tea from hip fracture [125,126].

The effectiveness of tea on fracture reduction was first reported on 1995 [125]. A total of 5618 women aged 50 years older living in Europe were enrolled in the Mediterranean Osteoporosis (MEDOS) Study to observe the risk factors for hip fracture. In all tea-drinking countries, a significant lower risk for hip fracture was observed [125]. The MEDOS Study enrolled 730 men with hip fracture in Europe. Drinking tea showed a 28% decrease in the risk of hip fracture [126].

In addition to fracture reduction, the association between BMD and tea drinking has been previously reported. Hoover et al. reported tea drinkers have higher lumbar and femoral BMDs and lean body mass in 62 postmenopausal women in Canada [122]. In a study on 1256 post-menopausal women in the United Kingdom, Hegarty et al. found drinking tea is associated with higher BMD at the lumbar spine and Ward’s triangle without affecting the femoral neck [121]. Similarly, 47,720 women aged 45–58 years participated in the Danish Osteoporosis Prevention Study (DOPS). The results indicated a protective effect of tea drinking in those with T-scores above −0.75 in the femoral neck [127]. Wu et al. reported an epidemiological survey on 497 men and 540 women in Taiwan. Tea drinkers were found to have higher lumbar BMD, while the duration of tea usage was the only independent factor for BMD [124]. Hossein-Nezhad et al. reported that female tea drinkers that consumed five cups or more daily had higher BMD in the hip among 830 men and women (20–76 years old) living in Iran [128]. In an investigation of the prevalence of postmenopausal osteoporosis in Turkey (IPPOT Study), 742 women were included in the study. Hamdi Kara et al. reported a higher T-score in BMD in those who drink tea [129]. Muraki et al. reported that BMD was higher in subjects with tea-drinking habits in a cross-sectional study of 632 women older than 60 years [130]. In a study with 354 subjects (151 normal and 203 osteoporotic) from India, drinking seven cups or more of tea per day was found to be a protective factor from osteoporosis [131].

There are several prospective studies regarding tea and BMDs. A four-year cross-sectional and prospective longitudinal study consisting of 1500 women aged 70–85 years in Australia found that tea drinkers had higher total hip BMD in the cross-sectional analysis and less total hip BMD loss in the prospective analysis [132]. In a prospective cohort study in Australia with 1188 women aged 75 years and older, Myers et al. showed that in senile women with high fracture risk, higher intake of some flavonoids and black tea can lower the risk of major osteoporotic and hip fracture-related hospitalization [133]. In the Women’s Health Initiative Observational Study with a multiethnic postmenopausal cohort (n = 91,465), tea consumption was associated with increased total body BMD. However, the fracture risk at the wrist and hip was not reduced by tea consumption [123]. Tavani et al. compared 279 women in Italy with hip fracture and 1061 controls. They found no effect of tea drinking on reducing the hip fracture risk after adjusting for the confounding factors including age, education, smoking status, menopausal status, total alcohol drinking, and calcium intake [134].

On the contrary, several studies could not show tea to be effective for BMD improvement or fracture reduction. In a case-control study examining 277 controls, 154 wrist fracture patients, and 102 hip fracture patients in Canadian women (50–84 years), there was no correlation between tea consumption and fracture risk [135]. In 703 unrelated elderly older than 90 years and 581 pairs of controls and hip fracture incident patients (71 ± 7 years) in China, there was no relationship between tea consumption and fracture risk [136,137]. On the contrary, tea drinkers showed greater hip fracture risk (OR 22.8; 95% CI 3.73 to 139.43) in a case-controlled study in an Indian urban population [138]. In a cross-sectional study with 1495 Chinese women, 1.9% higher BMD and 3.6% lower buckling ratio were found in tea drinkers (*n* = 732) compared to non-tea drinkers (*n* = 763). The results indicated that tea drinking enhanced bone strength in Chinese women [139]. The China Kadoorie Biobank (CKB) included 453,625 participants. Daily tea drinkers had lower risk of fracture (hazard ratio (HR): 0.88). Green tea drinkers (HR: 0.80) and those who had drank tea for 30 years or longer (HR: 0.68) had a reduced risk of hip fracture [140]. In a sub-analysis of CKB, 20,643 participants who finished a baseline survey (2004–2008) and re-survey (2013–2014) were included. Prolonged weekly tea consumption in women, but not men, was shown to result in higher BMD in calcaneus measures [141].

#### 2.3.2. Human Clinical Trials

Two randomized control trials were found. Shen et al. conducted a 6-month randomized placebo-controlled trial to evaluated the effects of green tea and Tai Chi (TC) on bone health in 171 postmenopausal osteopenia women. They found significant increases in BAP level and BAP/TRAP ratio due to GTP intake (at 1 month) and TC (at 3 months). At the end of study, muscle strength was significantly improved by GTP, TC, and GTP + TC interventions. There was no effect of GTP and TC on serum TRAP, serum and urinary calcium, and inorganic phosphate [142,143]. Improvement of muscle strength may decrease falls and eventually reduce fractures. Similar to the previous animal study, there was a significant reduction of urinary 8-OHdG, an oxidative DNA damage biomarker, concentrations in all three treated groups at 3 months and 6 months [144].

**Figure 1:**
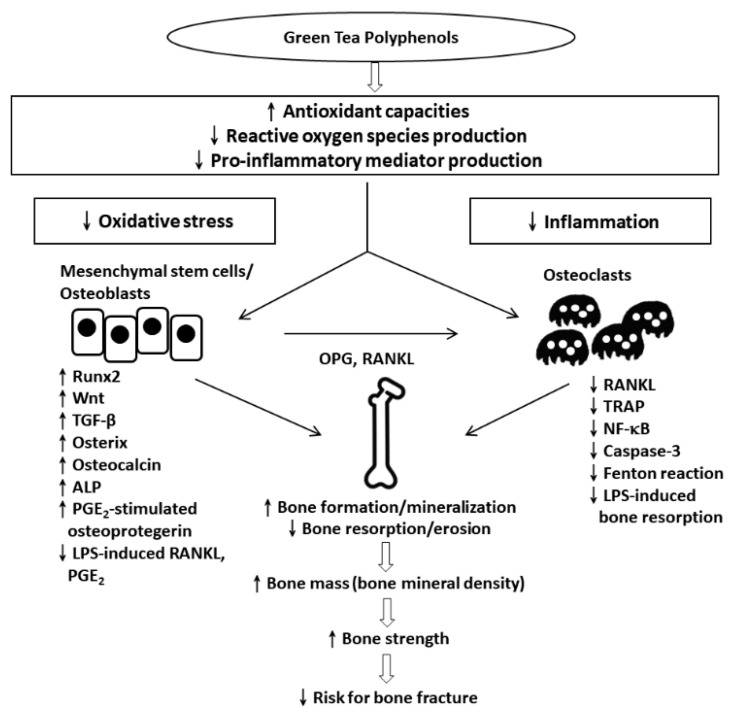
Summarized mechanisms of green tea polyphenols (GTP) in bone protection. Green tea catechins increase antioxidant capacities, reduce oxidative stress, and suppress the production of pro-inflammatory mediators. In osteoblasts, GTP inhibits oxidative stress by the upregulation of Runx2, Wnt, TGF-β, Osterix, Osteocalcin, and ALP. Moreover, RANKL, TRAP, and NF-κB activities are downregulated and caspase- 3 and Fenton reaction activities are upregulated in osteoclasts by GTP treatment. These actions should contribute to enhance bone formation/mineralization, and reduce bone resorption/erosion, resulting in increased bone mass, bone mineral density, and bone strength, leading to a reduced risk of bone fracture. ALP, alkaline phosphatase; GPX, glutathione peroxidase; NF-κB, nuclear transcription factor-κB; PGE2, prostaglandin E2; RANKL, receptor activator of nuclear transcription factor-κB ligand; Runx2, Runt-related transcription factor 2; SOD, superoxide dismutase; TGF-β, transforming growth factor β; TRAP, tartrate-resistant acid phosphatase; **↑**, upregulated; ↓, downregulated.

The Minnesota Green Tea Trial was a 12-month randomized, double-blind, placebo-controlled clinical trial with 937 50–70 years old postmenopausal women to treated placebo or GTE containing 843 mg (−)-epigallocatechin-3-gallate [145,146]. The effect of BMD was analyzed in a subgroup 121 high body mass index (BMI) (BMI ≥ 25.0) individuals. The differences in changes in percentage of body fat, total fat mass, BMI, or BMD over 12 months between 60 taking a placebo and 61 women taking GTE were not significant. In participants with higher BMI, GTE reduced gynoid and tissue fat percentage. There were no changes in circulating ghrelin, insulin, adiponectin, or leptin concentrations [147] (Table 3; Figure 1).

## 3. Conclusions and Prospective 

Osteoporotic fractures may cause morbidity and even mortality in the elderly [148,149,150,151,152]. Among osteoporotic fracture, hip fracture usually leads to hospitalization, disability, and even death [153,154,155,156]. Though there are considerable advances in the fracture risk assessment and the improvement of medication to decrease the risk of fragility fracture, many people with high fracture risk do not receive adequate investigation and treatment. Osteoporosis treatment takes a life-long effort. For treatment with bisphosphonates, oral forms may cause upper gastrointestinal adverse reactions and intravenous forms may lead to acute phase reaction, both of which are common. An atypical femoral shaft or subtrochanteric fractures and osteonecrosis of the jaw are rare adverse effects of bisphosphonates and RANKL inhibitors [157]. Vasomotor symptoms, muscle cramps, and venous thrombosis are adverse effects related to selective estrogen receptor modulators. Elevated urine or serum calcium, or higher serum uric acid or muscle cramps, are adverse effects related to parathyroid hormone receptor agonists [158,159]. Due to the long-term nature of osteoporosis treatment, many individuals fear adverse effects from the medications above. Thus, an agent that promotes bone health may be beneficial to individuals with osteoporosis, especially for senile patients.

GTE has been shown to have bone health-promoting effects in the studies reviewed in this paper. Improvement in muscle function may also contribute to fracture reduction, though many obstacles still exist in efficiently applying GTE as a nutrient to protect bone or prevent fracture. First, bioactive ingredients vary between studies, depending on the origin of tea, time of harvest, method of tea preparation and preservation, the batch of tea, stability of bioactive ingredients, and the route of administration in humans. These obstacles make it difficult to standardize bioactive ingredients. Therefore, it is almost impossible to repeat studies even in the same center once the same batch of bioactive ingredients runs out. This difficulty can be observed by the low number of randomized control trials published (two). In the two randomized control trials, only BMD or bone markers results were reported, and no data on fractures. In most phase III trials of currently available anti-osteoporotic drugs, sample sizes consist of several thousand subjects. If we want to test the efficacy of GTE in preventing fracture, at least ten thousand more subjects may be required, as well as the financial support of government or big pharmaceutical companies. Thus, a more cost-effective, alternative approach is required to test the efficacy of nutrients in preventing fracture.

The Minnesota Green Tea Trial is a well-focused trial because only EGCG was used. Capsule EGCG also simplifies the bioactive ingredients. Since EGCG is very unstable, it is important to protect its bioactivity in capsule form. Treatment duration is also important. In the Minnesota Green Tea Trial, treatment duration was only one year. Though many anti-osteoporotic medications show great efficacy in reducing fracture one year after treatment, it is not easy for naturally-derived nutrients to achieve similar effects to clinical drugs. Dose is also an important issue. In the Minnesota Green Tea Trial, 800 mg EGCG daily was used for one year. It is unknown whether this dose is suitable for protecting bone health. Since both anabolic and anti-catabolic effects of EGCG were found in MSC and stromal cells [37,39,40,41,56,57,58], pre-osteoclasts may be the target cells of EGCG. The anti-catabolic effects of EGCG can vary depending on the MSC/stromal cell regulation of osteoclastogenesis at lower concentrations, or on the direct inhibition of osteoclastogenesis at higher concentrations.

Even though randomized control trials did not show that GTE provides anti-fracture efficacy, safety data in the trials are promising. With the current safety data available, GTE should be relatively safe for promoting bone health. More longitudinal studies are required to prove its safety for long-term use. On the basis of the currently available results, other results that are related to bone health, such as quality of life, balance, function, and mobility, may be a good alternative way to evaluate the beneficial effects of GTE. Due to limited of catechins in human data on BMD and anti-fracture efficacy, a large-scale, long-term, placebo-controlled randomized trial with a tea intervention of optimal duration is required for determining anti-fracture efficacy.

## Figures and Tables

**Table 1:** In vitro effects of catechins.

First Author, Year	Experimental Design and Treatments	Results
Nakagawa, 2002 [52]	Model: primary cultured Crude murine osteoclast-like multinucleated cells (OCLs) Treatments: EGCG (25–100 μM) for 24 h	EGCG treatments: ↑ apoptotic cell death of osteoclast-like multinucleated cells, whereas osteoblasts affected the Fenton reaction primarily involved in EGCG-induced osteoclastic cell death
Chen, 2003 [60]	Model: The pluripotent mesenchymal cell, D1, cloned from mouse bone marrow cells Treatments: EC, ECG, EGC, and EGCG at a concentration of 1 μM, the achievable concentration with one cup of tea drinking, 10 μM and 100 μM for 24 and 48 h, respectively.	Compared to vehicle group, EGCG groups: ↑ mRNA expression of OPG
Yun, 2004 [53]	Model: co-culture system of mouse bone marrow cells and calvarial primary osteoblastic cells Treatments: EGCG (20 μM) in the presence of sonicated *P. gingivalis* extracts.	EGCG treatments: ↓expression of MMP-9 mRNA ↓ osteoclast formation
Chen, 2005 [64]	Model: The pluripotent mesenchymal cell, D1, cloned from mouse bone marrow cells Treatments: vehicle, EGCG (1 and 10 μM) for 48 h	Compared to vehicle group, EGCG groups: ↑ mRNA expression of Runx2, osterix, osteocalcin, ALP after 48 h ↑ ALP activity after 4 d, 7 d, and 14 d ↑ mineralization after 2–4 w ↓ thymidine incorporation
Tokuda, 2007 [55]	Model: osteoblastic cell line MC3T3-E1 Treatments: EGCG in a dose-dependent manner in the range between 1 and 100 μM	EGCG treatments: ↓ ET-1-induced IL-6 synthesis ↔ ET-1-induced phosphorylation of p38 MAP kinase ↓ phosphorylation of p44/p42 MAP kinase and 12-O-tetradecanoylphorbol 13-acetate (TPA), a direct activator of PKC induced by ET-1
Tokuda, 2007 [56]	Model: osteoblastic cell line MC3T3-E1 Treatments: EGCG in a dose-dependent manner in the range between 1 and 100 μM	EGCG treatments: ↑ PGF(2alpha)-induced VEGF synthesis ↔ PGF(2alpha)-induced phosphorylation of p44/p42 MAP kinase ↑ phosphorylation of SAPK/JNK induced by PGF(2alpha) ↔ PGF(2alpha)-induced phosphorylation of p38 MAP kinase ↑ PGF(2alpha)-induced phosphorylation of c-Jun
Vali, 2007 [67]	Model: the formation of mineralized bone nodules by SaOS-2 human osteoblast (HOB)-like cells Treatments: EGCG at concentrations of 1–5 μM for 48 h	EGCG treatments: ↑ number and area of mineralized bone nodules ↑ alkaline phosphatase activity ↓ protein levels of Runx2
Hayashi, 2008 [71]	Model: osteoblast-like MC3T3-E1 cells Treatments: the cells were pretreated with various doses of EGCG (0, 10, and 30 μM) for 48 h	EGCG treatments: ↓ HSP27 induction stimulated by TGF-β ↔ HSP70 levels↔ advanced oxidation protein products ↔ TGF-β-induced phosphorylation of p38 MAP kinase and ERK1/2 ↓ phosphorylation of both MKK4 and TAK1 induced by TGF-β ↓ TGF-β-induced phosphorylation of SAPK/JNK without affecting the phosphorylation of Smad2
Morinobu, 2008 [57]	Model: Mononuclear cells were isolated from peripheral blood obtained from healthy donors. Treatments: For osteoclast differentiation, the cells were cultured in the presence of M-CSF (50 ng/mL) and RANKL (100 ng/mL) for 6 d EGCG or other chemicals were added throughout the culture, and half the medium was replaced every 2–3 d	EGCG treatments: ↓ generation of TRAP-positive multinucleated cells, bone resorption activity, and osteoclast-specific gene expression ↓ expression of nuclear factor of activated T cells c1 (NF-ATc1), but not of NF-κB, c-Fos, and c-Jun ↔ cell viability
Takai, 2008 [66]	Model: osteoblast-like MC3T3-E1 cells; primary-cultured osteoblasts were obtained from the calvaria of newborn (1 or 2-day old) BALB/c mice Treatments: EGCG (0–30 μM) for 24 h	EGCG treatments: ↓ IL-6 synthesis and IL-6 mRNA expression stimulated by PDGF-BB ↓PDGF-BB-induced phosphorylation of SAPK/JNK ↔ levels of osteocalcin and osteoprotegerin in MC3T3-E1 cells ↔ PDGF-BB-induced autophosphorylation of PDGF receptor β ↔ PDGF-BB-induced phosphorylation of p44/p42 MAP kinase and p38 MAP kinase ↔ PDGF-BB-induced phosphorylation of Akt and p70 S6 kinase
Tokuda, 2008 [70]	Model: osteoblast-like MC3T3-E1 cells Treatments: EGCG (0–30 μM) for 24 h	EGCG treatments: ↓ IL-6 synthesis stimulated by FGF-2 in a dose-dependent manner ↓ FGF-2-induced phosphorylation of p44/p42 MAP kinase and p38 MAP kinase
Lin, 2009 [59]	Model: murine preosteoclast cells (RAW 264.7); bone marrow macrophages (BMMs) Treatments: RANKL (100 ng/mL), RANKL+EGCG (0, 10, 20, 50, and 100 μM) for 24 h	Compared to RANKL group, EGCG treatment: ↓ differentiation of osteoclasts and the formation of pits ↓ RANKL-induced NF-κB transcriptional activity and nuclear translocation
Lee, 2010 [58]	Model: bone marrow macrophages (BMMs) Treatments: Pretreatment with EGCG under RANKL-induced osteoclastogenesis	EGCG treatments: ↓ RANKL-induced the gene expression of c-Fos and nuclear factor of activated T-cells (NFATc1) ↓ RANKL-induced activation of c-Jun N-terminal protein kinase (JNK) pathway, among the three well known mitogen-activated protein kinases ↓ RANKL-induced phosphorylation of the NF-κB p65 subunit at Ser276 and NF-κB transcriptional activity ↔ degradation of IκBα and NF-κB DNA-binding in BMMs
Oka, 2012 [54]	Model: rat osteoclast precursors cells and mature osteoclasts Treatments: vehicle, EGCG (10 and 100 μM) for 12 or 24 h	Compared to vehicle group, EGCG groups: ↓ formation and differentiation of osteoclasts via inhibition of MMPs ↓actin ring formation
Tominari, 2015 [63]	Model: Primary osteoblastic cells were isolated from newborn mouse calvariae Treatments: cells were treated with LPS (1μg/mL) with and without EGCG (30, 60, or 90 μM), and further cultured for 24 h for measurement of PGE2	EGCG treatments: ↓LPS-induced expression of RANKL mRNA in osteoblasts at 12–24 h ↓LPS-induces PGE_2_ production in osteoblasts at 12–24 h
Qiu, 2016 [79]	Model: human bone marrow-derived MSCs	EGCG treatments: ↑miR-210 ↑RUNX2, BMP-2, ALP, and PINP
Kuroyanagi, 2017 [62]	Model: Cloned osteoblast-like MC3T3-E1 cells Treatments: cells were pretreated with EGCG (0, 10, 30, and 50 μM) for 60 min, and then stimulated by 10 μM of PGE_2_	EGCG treatments: ↑PGE_2_-stimulated mRNA and protein expression of osteoprotegerin 48 h after treatment
Lin, 2018 [65]	Model: human bone marrow stem cells Treatments: EGCG with concentrations of 1 μM and 10 μM	Compared to control group, EGCG groups: ↑ mRNA expression of BMP2, Runx2, alkaline phosphatase (ALP), osteonectin, and osteocalcin 48 h after treatment ↑ ALP activity both 7 and 14 days after treatment. ↑ mineralization two weeks after treatment
Chen, 2019 [61]	Model: Murine bone marrow stromal ST2 cells; Murine RAW 264.7 cells Treatments: Cells were treated by 1 μM and 10 μM of EGCG	Compared to control group, EGCG groups: ↓ RANKL/OPG ratio in both mRNA expression and secretory protein levels ↓ osteoclastogenesis via the RANK/RANKL/OPG pathway

ALP, alkaline phosphatase; BMD, Bone mineral density; BMP-2, Bone Morphogenetic Protein 2; BR, buckling ratio; COX, cyclooxygenase; EGCG, epigallocatechin gallate; FHP, free hydroxyproline; GAG, glycosaminoglycan; GT, green tea; GTE, green tea extract; GTP, green tea polyphenols; iNOS, inducible nitric oxide synthase; IL-1β, interleukin-1β; LPS, lipopolysaccharide; MREI, molar roots exposure index; NANA, N-acetylneuraminic acid; NF-κB, nuclear factor kappa-B; NO, nitric oxide; RANKL, Receptor activator of nuclear factor kappa-Β ligand; RUNX2, Runt-related transcription factor 2; OPG, osteoprotegerin; OVX, ovariectomized; PGE2, prostaglandin E2; SG, salivary glands; TC, Tai Chi; TNF-α, tumor necrosis factor-α;↑, increase; ↓, decrease; ↔, no change.

**Table 2:** In vivo effects of catechins.

First Author, Year	Experimental Design and Treatments	Results
Shen, 2008 [86]	Model: a mouse model of OVX-induced bone loss 14-mo-old female rats (*n* = 10/group) Treatments: A 16-week study of a 2 (SHAM vs. OVX) × 3 (no GTP, 0.1% GTP, and 0.5% GTP in drinking water) factorial design	↔ femur bone mineral density between baseline and the SHAM+0.5% GTP group. In OVX group: ↓ liver glutathione peroxidase activity, serum estradiol, and bone mineral density group. GTP supplementation: ↑ urinary epigallocatechin and epicatechin concentrations, liver glutathione peroxidase activity, and femur bone mineral density decreased urinary 8-hydroxy-2’-deoxyguanosine and urinary calcium levels. ↔ serum estradiol and blood chemistry levels.
Iwaniec, 2009 [108]	Model: a mouse model of obese Male leptin-deficient (ob/ob) obese mice and male C57Bl/6 WT littermates (4-week-old) Treatments: Each group had equal numbers of obese and lean mice. Groups included 0% GTE (control), 1% GTE, and 2% GTE for 6 w	Compared to control group, GTE groups: Neither genotype affected femoral bone mineral density ↓ femur length, volume, mineral content, cortical volume, cortical thickness, cancellous bone volume/tissue volume, and trabecular thickness in lumbar vertebrae in GTE groups.
Shen, 2009 [87]	Model: a mouse model of OVX-induced bone loss 14-month-old female rats Treatments: A 16-w study of 2 (SHAM vs. OVX) × 3 (no GTP, 0.1% GTP, and 0.5% GTP in drinking water) factorial design	GTP supplementation: ↑ trabecular volume, thickness, number, and bone formation of proximal tibia, periosteal bone formation rate of tibia shaft, and cortical thickness and area of femur ↓ trabecular separation and bone erosion of proximal tibia, and endocortical bone eroded surface of tibia shaft
Shen, 2010 [98]	Model: a mouse model of LPS-induced chronic inflammation and bone loss Virgin CD female rats (3 months old) Treatments: placebo implantation (P), lipopolysaccharide (LPS) administration (L), P+0.5% GTP (PG) and LPS+0.5% GTP (LG) for 12 w 2 (placebo vs. LPS administration) × 2 (no GTP vs. 0.5% GTP in drinking water) factorial design	Neither LPS administration nor GTP levels affected body weight and femoral bone area throughout the study period. LPS administration: ↓ femur BMC and BMD, and serum OC levels ↑ serum TRAP, urinary 8-OHdG, and spleen mRNA expression of TNF-α and COX-2 levels GTP supplementation: ↑values for femur BMC, BMD, and serum OC ↓values for serum TRAP, urinary 8-OHdG, and spleen mRNA expression of TNF-α and COX-2 levels
Shen, 2010 [100]	Model: a mouse model of LPS-induced chronic inflammation and bone loss Virgin 3-month-old CD female rats Treatments: Among the LPS treated rats, a 2 (no GTP vs. 0.5% GTP in drinking water) × 2 (0 vs. 0.05 μg/kg body weight 1-α-OH-vitamin D3) factorial design enabled evaluation of effects of GTP, 1-α-OH-vitamin D3, and GTP × 1-α-OH-vitamin D3 interaction on chronic inflammation-induced bone loss along with related mechanism(s). In addition, a group receiving placebo administration only (the P group) was used.	LPS administration: ↓ Values for bone mass ↑ Values for serum tartrate-resistant acid phosphatase (TRAP), urinary 8-hydroxy-2′-deoxyguanosine, and mRNA expression of tumor necrosis factor-α and cyclooxygenase-2 in spleen GTP supplementation: ↑ urinary epigallocatechin and epicatechin concentrations A synergistic effect of GTP and alphacalcidol was observed in these parameters Neither GTP nor alphacalcidol affected femoral bone area or serum osteocalcin.
Shen, 2011 [99]	Model: a mouse model of LPS-induced chronic inflammation and bone loss 3-month-old virgin Sprague Dawley (SD) female rats Treatments: placebo implantation (P), lipopolysaccharide (LPS) administration (L), P+0.5% GTP (PG), or LPS+0.5% GTP (LG) for 12 w 2 (placebo vs. LPS administration) × 2 (no GTP vs. 0.5% GTP in drinking water) factorial design	LPS group: ↓ trabecular volume fraction, thickness, and bone formation in proximal tibia ↑osteoclast number and surface perimeter in proximal tibia and eroded surface in endocortical tibial shafts GTP group: ↑ trabecular volume fraction and number in both femur and tibia and periosteal bone formation rate in tibial shafts ↓trabecular separation in proximal tibia and eroded surface in endocortical tibial shafts ↑ strength of femur ↓ TNF-α expression in tibia
Shen, 2011 [109]	Model: a mouse model of LPS-induced chronic inflammation Virgin CD female rats (3 months old) Treatments: (1) LPS administration (L, *n* = 10), (2) LPS + 1-α-OH-vitamin D3 (LD, *n* = 10), (3) LPS + GTP (LG, *n* = 10), and (4) LPS + GTP + 1-α-OH-vitamin D3 (LGD, *n* = 10) for 12 w	Compared to LPS group, Both GTP and alfacalcidol: ↑ femoral mass, trabecular volume, thickness, and number in proximal tibia and femur, and periosteal bone formation rate in tibial shafts ↓ trabecular separation and osteoclast number in proximal tibia and eroded surface in endocortical tibial shafts ↑ femoral strength ↓ TNF-α expression in proximal tibia
Shen, 2011 [88]	Model: a mouse model of orchidectomy-induced male osteoporosis Virgin male F344 rats (15 months old) Treatments: A 2 (sham vs. orchidectomy) × 2 (no GTP and 0.5% GTP in drinking water) factorial design was studied for 16 w	Compared to orchidectomy group, GTP supplementation: ↑ serum osteocalcin concentrations, bone mineral density, and trabecular volume, number, and strength of femur ↑ trabecular volume and thickness and bone formation in both the proximal tibia and periosteal tibial shaft ↓ eroded surface in the proximal tibia and endocortical tibial shaft ↓ liver glutathione peroxidase activity
Oka, 2012 [54]	Model: cultures of rat osteoclast precursors cells and mature osteoclasts Treatments: the black tea polyphenol, theaflavin-3,3’-digallate (TFDG), or EGCG (10 and 100 µM) was added	Compared to control group, TFDG or EGCG treatment: ↓ numbers of multinucleated osteoclasts and actin rings ↓ MMP-2 and MMP-9 activities ↓ MMP-9 mRNA levels
Shen, 2012 [101]	Model: a mouse model of high-fat (HF) diet-induced obese female 3-month-old Sprague Dawley female rats Treatments: After low-fat (LF) (10% energy as fat) (*n* = 12) or HF diet (45% energy as fat) (*n* = 24) ad libitum for 4 m, whereas those in the HF diet group were randomly divided into two groups: with (the HF + GTP group, *n* = 12) or without GTP (HF group, *n* = 12) in drinking water, in addition to an HF diet for another 4 m	Compared to HF group, GTP supplementation: ↑ percentage of fat-free mass, bone mineral density and strength, and GPX protein expression ↓ percentage of fat mass, serum insulin–like growth factor I, leptin, adiponectin, and proinflammatory cytokines in the obese rats
Chen, 2013 [94]	Model: a mouse model of OVX-induced bone loss Twelve-week-old female Sprague-Dawley rats Treatments: (1) sham-operated controls (SHAM; *n* = 8); (2) ovariectomized controls (OVX; *n* = 14); (3) OVX with EGCG 0.34 mg/kg/day (OVX + 1 EGCG; *n* = 12; estimated peak serum concentration, 1 μM); and (4) OVX with EGCG 3.4 mg/kg/day (OVX + 10 EGCG; n = 14; estimated peak serum concentration, 10 μM). Three months after OVX, EGCG was given intraperitoneally for 12 w	Compared to untreated control group, EGCG treatment: ↑ bone volume, trabecular thickness, trabecular numbness, and trabecular separation. Similar ↑ in bone volume and trabecular thickness in third lumbar spine. ↑ bone volume in tibial cortex ↑ trabecular number and trabecular volume in histology
Shen, 2013 [102]	Model: a mouse model of high-fat diet (HFD)-induced obese female 3-month-old virgin Sprague-Dawley female rats Treatments: After 4 m of HF diet, they were randomly divided into two groups, with GTP supplement in drinking water (the HFD + GTP group, *n* = 12) or without GTP (the HFD group, *n* = 12), in addition to the same HFD for another 4 m	Compared to HF group, GTP supplementation: ↑ BMD at the femur, a greater trabecular volume, thickness, and number at the proximal tibia, a larger cortical area and thickness at the tibial shaft, and a greater trabecular volume and thickness at the femur and the lumbar vertebrae ↓Tb.Sp, MAR, bone formation rate, and eroded surface at the tibia
Yoshinaga, 2014 [104]	Model: a mouse model of LPS-induced PE 9-week-old male Lewis rats Treatments: LPS group (*n* = 12); the green tea extract group (*n* = 12); and the phosphate buffered saline (PBS) group (*n* = 6) for 20 d	Compared to LPS group, GTE group: ↓loss of attachment, level of alveolar bone, inflammatory cell infiltration and RANKL expression
Cai, 2015 [105]	Model: a mouse PE model induced by P. gingivalis infection Female BALB/c mice (8-w-old) Treatments: EGCG group (0.02%) or drinking water group both infected with *P. gingivalis* every 2 days for 15 w	Compared to water group, EGCG group: ↓ reduction in bone loss ↓ inflammatory serum mediators ↓ high positive areas of IL-17 and IL-1β ↓ IL-1β, IL-6, IL-17, TNF-α, and other mediators, but not IL-23
Gennaro, 2015 [106]	Model: a mouse models of type 1 diabetes Male Wistar rats (8–10-w-old) Treatments: Groups included the Diab group (type 1 diabetes) and Ctr (control group). Each group was further divided into two control groups (water and green tea-treated) and two diabetic groups (water and green-tea treated) measured at 15, 30, 60, and 90 d	↓ number of cells expressing RANKL and TNF-α in diabetic rats treated with green tea. ↑ cells positive for OPG, RUNX-2, and IL-10 in diabetic rats
Shen, 2015 [89]	Model: obese rats fed a high-fat diet (HFD) or a caloric restricted diet (CRD) Sprague Dawley female rats (3-mo-old) Treatments: rats were fed with an HFD diet ad libitum for 4 m Then, based on body weight, the animals were assigned to one of the four groups ((HFD vs CRD with 35% caloric deficit) × (0% vs 0.5% GTP in drinking water)) in a two-factorial study for another 4 m	In CRD: ↓ percent fat mass; bone mass and trabecular number of tibia, femur, and lumbar vertebrae; femoral strength; trabecular and cortical thickness of tibia; insulin-like growth factor-I and leptin ↑ percent fat-free mass; trabecular separation of tibia and femur; eroded surface of tibia; bone formation rate and erosion rate at tibia shaft; and adiponectin GTP supplementation: ↑ femoral mass and strength (*p* = 0.026), trabecular thickness (*p* = 0.012) and number (*p* = 0.019), and cortical thickness of tibia (*p* < 0.001) ↓ trabecular separation (*p* = 0.021), formation rate (*p* < 0.001), eroded surface (*p* < 0.001) at proximal tibia, and insulin-like growth factor-I and leptin. There were significant interactions (diet type × GTP) on osteoblast surface/bone surface, mineral apposition rate at periosteal and endocortical bones, periosteal bone formation rate, and trabecular thickness at femur and lumbar vertebrate (*p* < 0.05).
Tominari, 2015 [63]	Model: LPS-induced alveolar bone loss in mice Treatments: injected LPS with or without EGCG (0.5 mg/mouse) into the gingiva of the lower mandibles of mice. After seven days of the first injection, alveolar bone was collected from mouse	EGCG treatment ↓ LPS-induced bone resorption and alveolar bone loss in mice
Liu, 2018 [97]	Model: a mouse model of steroid-induced bone loss Female experimental SD rats (8-week-old) Treatments: control group, dexamethasone (DEX) groups, and DEX with EGCG (5 mg/kg/day)	↑Osteoblast cell viability, ALP, and SOD activities ↑11β-HSD activity ↑Nrf2/HO-1 Signaling ↓DEX-Induced Oxidative Stress, DEX-Induced apoptosis of Osteoblasts ↑Osteogenic differentiation in primary osteoblasts ↑Improve microarchitecture
Xi, 2018 [91]	Model: a mouse model of dexamethasone-induced osteoporosis Male C57BLKS/J mice (6-week-old) Treatments: Groups included control, model, and EGCG (0.5 mg/kg/day) for 4 weeks. Model and EGCG groups were injected with dexamethasone (5 mg/kg/day) to establish the osteoporosis model.	↓ serum calcium, urinary calcium, body weight, and body fat ↑ leptin in mice with secondary osteoporosis Inhibited structure score of articular cartilage and cancellous bone in proximal tibia metaphysis in mice with secondary osteoporosis. ↓ alkaline phosphatase activity, runt related transcription factor 2, and osterix mRNA expression. ↑ protein expression of cyclin D1, Wnt, and β catenin. ↓ peroxisome proliferator activated receptor γ protein expression in mice with secondary osteoporosis.
de Almeida, 2019 [46]	Model: a mouse model of ligature-induced experimental periodontitis (EP) Male Wistar rats (3-month-old) Treatments: control (no EP), EP (ligature induction), SRP (SRP given after 7 d of EP), and SRP/GT (SRP and GTE given after 7 d of EP), measured at 14, 22, and 37 d.	Compared to control group, SRP/GT group: ↓ inflammatory process ↓ immunolabeling pattern of IL-1ß and TNF-α ↑ immunolabeling pattern of IL-10 ↓ TRAP-positive multinucleated osteoclasts ↑ PBF
Lin, 2019 [96]	Model: A defect on left distal femur was created by using a 0.5 mm cone-shape hand drill Male Sprague-Dawley (SD) rats aged 12 w Treatments: vehicle and EGCG were applied locally by percutaneous local injection 2 d after defect creation for 2 w	Compared to control group, EGCG groups: ↑ de novo bone formation by increasing bone volume ↑ mechanical properties including max load, break point, stiffness, area under the max load curve, area under the break point curve, and ultimate stress
Lin, 2020 [95]	Model: a mouse model of tibia fracture Male Sprague–Dawley (SD) rats at 12 weeks Treatments: vehicle treatment as control group (Ctrl) (*n* = 28) and fracture with treatment of EGCG (EGCG) (*n* = 28). EGCG, 40 μL at 10 μM, with a total dose of 0.52 μg/kg/time treated daily with EGCG or vehicle by percutaneous local injection for 2 w	Compared to control group, EGCG groups: ↑ callus formation by increasing the bone volume ↑ mechanical properties of the tibial bone, including the maximal load, break load, stiffness, and Young’s modulus ↑ bone matrix formation and stronger expression of BMP-2

ALP, alkaline phosphatase; BMD, Bone mineral density; BMP-2, Bone Morphogenetic Protein 2; BR, buckling ratio; COX, cyclooxygenase; EGCG, epigallocatechin gallate; FHP, free hydroxyproline; GAG, glycosaminoglycan; GT, green tea; GTE, green tea extract; GTP, green tea polyphenols; iNOS, inducible nitric oxide synthase; IL-1β, interleukin-1β; LPS, lipopolysaccharide; MREI, molar roots exposure index; NANA, N-acetylneuraminic acid; NF-κB, nuclear factor kappa-B; NO, nitric oxide; RANKL, Receptor activator of nuclear factor kappa-Β ligand; RUNX2, Runt-related transcription factor 2; OPG, osteoprotegerin; OVX, ovariectomized; SG, salivary glands; TC, Tai Chi; TNF-α, tumor necrosis factor-α; ↑, increase; ↓, decrease; ↔, no change.

**Table 3:** Human effects of catechins.

First Author, Year	Experimental Design and Treatments	Results
Kreiger, 1992 [135]	Model: a case-control study examined the effect of diet on the risk of postmenopausal fracture of the hip and wrist. Treatments: Cases, women aged 50–84 y, were admitted to one of four Metropolitan Toronto hospitals during the period September 1983 through May 1985. Controls were women of the same age, admitted to the same hospitals, and seen for orthopedic or general surgical complaints.	Coffee and tea consumption appeared to be unrelated to fracture risk.
Johnell, 1995 [125]	Model: a multicenter study to determine common international risk factors for hip fracture in women aged 50 y or more. Treatments: Women aged 50 y or more selected from the neighborhood or population registers served as controls. Cases and controls were interviewed using a structured questionnaire on work, physical activity, exposure to sunlight, reproductive, gynecologic status and history, height, weight, mental score, and consumption of tobacco, alcohol, calcium, coffee, and tea.	Significant risk factors identified by univariate analysis included low body mass index (BMI), short fertile period, low physical activity, lack of sunlight exposure, low milk consumption, no consumption of tea, and a poor mental score. A late menarche, poor mental score, low BMI and physical activity, low exposure to sunlight, and a low consumption of calcium and tea remained independent risk factors after multivariate analysis, accounting for 70% of hip fractures.
Tavani, 1995 [134]	Model: 279 cases of hip fracture and 1061 controls in hospital for acute, nonneoplastic nontraumatic, and non-hormone-related diseases Treatments: consumption of coffee and other methylxanthine-containing beverages	↔ hip fractures
Hoover, 1996 [122]	Model: Physical and lifestyle data were collected from 62 postmenopausal women who had declined hormone replacement therapy. Treatments: Tea drinking was assessed by self-completed questionnaire and women were categorized as tea drinkers or non-tea drinkers.	Compared to non-tea drinkers, tea drinkers: ↑ associated with both bone density ↑ femoral BMD, lumbar BMD, and lean body mass
Kanis, 1999 [126]	Model: a multicenter study to identify risk factors for hip fracture in men aged 50 y or more. Treatments: 730 men with hip fracture from 14 centers, and 1132 age-stratified controls selected from the neighborhood or population registers. questionnaire examined aspects of work, physical activity past and present, diseases and drugs, height, weight, indices of co-morbidity, and consumption of tobacco, alcohol, calcium, coffee, and tea.	Of the potentially ‘reversible’ risk factors, BMI, leisure exercise, exposure to sunlight, and consumption of tea and alcohol and tobacco remained independent risk factors after multivariate analysis, accounting for 54% of hip fractures
Hegarty, 2000 [121]	Model: measured BMD at the lumbar spine, femoral neck, greater trochanter, and Ward’s triangle in 1256 free-living women aged 65–76 y in Cambridge, United Kingdom. Treatments: Tea drinking was assessed by self-completed questionnaire and women were categorized as tea drinkers or non-tea drinkers	Compared to non-tea drinkers, tea drinkers: ↑ mean BMD measurements, adjusted for age and body mass index, at the lumbar spine (0.033 g/cm^2^; *p* = 0.03), greater trochanter (0.028 g/cm^2^; *p* = 0.004), and Ward’s triangle (0.025 g/cm^2^; *p* = 0.02). ↔ Differences at the femoral neck (0.013 g/cm^2^)
Vestergaard, 2001 [127]	Model: to predict spinal and femoral bone mineral density (BMD) in perimenopausal women from simple clinical and biochemical variables. Treatments: 2016 women 3–24 months after last menstrual bleeding. Mean age 50.1 ± 2.8 years. Independent factors: age, height, weight, number of full-term pregnancies, weekly hours of physical activity, sunbathing habits, use of sun bed, daily intake of calcium and vitamin D, smoking habits, and consumption of alcohol, coffee, and tea.	Conclusions: Simple clinical and biochemical variables are not useful to predict spinal and femoral BMD in the individual perimenopausal woman
Wu, 2002 [124]	Model: an epidemiological survey study Treatments: 497 men and 540 women, 30 y and older. All subjects were questioned about their habit of tea consumption and other lifestyle characteristics by means of a structured questionnaire.	Compared to non-tea drinkers, tea drinkers: ↑ lumbar spine BMDs the duration of tea consumption was the only independent determinant for the BMDs
Chen, 2003 [123]	Model: investigating associations of habitual drinking of regular tea with bone mineral density and fracture risk. Treatments: Study participants were a multiethnic postmenopausal cohort (*n* = 91,465) from the nationwide Women’s Health Initiative Observational Study. These women were recruited in the United States and aged 50-79 years at the time of enrollment (1994–1998). The average follow-up time was 4.1 y. Habitual consumption of regular tea was assessed with a structured questionnaire at baseline.	Multivariate analyses suggested a positive trend of increased total body bone mineral density with tea drinking (*p* < 0.05). Cox proportional hazard models did not show any significant association between tea drinking and the risk of fractures at the hip and forearm/wrist.
Devine, 2007 [132]	Model: Using both cross-sectional and longitudinal study designs, we examined the relation of tea consumption with hip structure. Treatments: Randomly selected women (*n* = 1500) aged 70–85 y participated in a 5-y prospective trial to evaluate whether oral calcium supplements prevent osteoporotic fractures.	In the cross-sectional analysis, tea drinkers: ↑ total hip areal bone mineral density (aBMD) In the prospective analysis over 4 y, tea drinkers: ↓ loss of total hip aBMD
Hossein-Nezhad, 2007 [128]	Model: BMD was measured at the lumbar spine and hip, in 830 men and women living in Tehran, all aged between 20 and 76 y. Treatments: The degree of tea consumption was assessed by questionnaire, and subjects were categorized as either tea drinkers (more than five cups of tea per day) or non-tea drinkers (equal to or less than five cups of tea per day)	Compared to non-tea drinkers, tea drinkers: ↑ BMD in the hip of female tea drinkers
Hamdi Kara, 2007 [129]	Model: investigation of prevalence of postmenopausal osteoporosis in Turkey (IPPOT Study) Treatments: 742 women were included in the study. The mean age was 57.6 ± 9.6 y, and mean age at natural menopause was 46.4 ± 5.6 y. A semi-structured questionnaire was completed by face-to-face interview, consisting of closed- and open-ended questions about demographic characteristics, nutritional status, and habits with two or more choices as possible responses.	Compared to non-tea drinkers, tea drinkers: ↑ T-scores, and BMD
Muraki, 2007 [130]	Model: to identify lifestyle factors associated with BMD. Treatments: A total of 632 women age > or = 60 y enrolled in this study. Subjects were interviewed about their lifestyle by means of a questionnaire regarding the consumption pattern of dietary items	Compared to smoking and cheese consumption, green tea drinking: ↑ BMD
Keramat, 2008 [131]	Model: a multicenter interview-based study conducted in selected hospitals and health centers from urban areas in Iran and India. Sample sizes included a total of 363 subjects from Iran (178 osteoporotic and 185 normal) and a total of 354 subjects from India (203 osteoporotic and 151 normal). Treatments: The case group included postmenopausal osteoporotic women, and the controls were chosen from postmenopausal women with normal bone density	Compared to non-tea drinkers, tea drinkers: ↑ significant protective factors in Iran.
Jha, 2010 [138]	Model: a case control investigation comprising 100 case subjects (57 women and 43 men) admitted with a first hip fracture into one of three hospitals across New Delhi. Treatments: The 100 controls were age- and sex-matched subjects who were either healthy visitors not related to the case patients or hospital staff. Information from all subjects was obtained through a questionnaire-based interview.	Tea and other caffeinated beverages were significant risk factors. Tea drinkers: ↑ risk of hip fracture (OR 22.8; 95% CI 3.73-139.43)
Du, 2011 [136]	Model: a cross-sectional study conducted in Dujiangyan Sichuan China. Treatments: 703 unrelated Chinese nonagenarians and centenarians (67.76% women, mean age 93.48 y) resident in Dujiangyan. Medical history of osteoporosis and the statement of fracture and habits (current and former) of smoking, alcohol consumption, tea consumption, and exercise were collected.	In summary, among nonagenarians and centenarians, among habits (current and former) of smoking, alcohol consumption, tea consumption, and exercise, there seems to be significant association of osteoporotic fracture only with current or former habits of alcohol consumption, former habit of exercise.
Shen, 2010 [143] Shen, 2012 [142] Qian, 2012 [144]	Model: a 6-month randomized placebo-controlled trial. Postmenopausal women with osteopenia received green tea polyphenols (GTP) supplement and/or Tai Chi (TC) exercise for 6 months. Treatments: A total of 171 postmenopausal osteopenic women were randomly assigned to four groups: (1) placebo (500 mg starch/day), (2) GTP (500 mg GTP/day), (3) placebo + TC (placebo plus TC training at 60 min/session, three sessions/week), and (4) GTP + TC (GTP plus TC training).	Compared to control group, GTP intake, and TC groups: ↑ bone-specific alkaline phosphatase (BAP) level ↑ BAP/TRAP ratio ↑ muscle strength ↔ serum TRAP, serum and urinary calcium, and inorganic phosphate Compared to control placebo group, GTP groups: ↓ urinary 8-OHdG concentrations ↓ oxidative damage biomarker
Zeng, 2013 [137]	Model: Face-to-face interviews to examined the association of dietary patterns with the risk of hip fractures in elderly Chinese. Treatments: A total of 581 pairs of hip fracture incident cases and controls (71 ± 7 y) were studied.	No significant association was found between the traditional dietary pattern (with a high intake of Chinese herbal tea, double stewed soup, processed meat and fish, and organ meat) and hip fracture risk.
Myers, 2015 [133]	Model: A total of 1188 women were assessed for habitual dietary intake with a food-frequency and beverage questionnaire. Treatments: Incidence of osteoporotic fracture requiring hospitalization was determined through the Western Australian Hospital Morbidity Data system	In comparison with the lowest tea intake category (≤1 cup/wk), consumption of ≥3 cups/d: ↓ 30% in the risk of any osteoporotic fracture compared with women in the lowest tertile of total flavonoid intake (from tea and diet): ↓risk of any osteoporotic fracture (HR: 0.65; 95% CI: 0.47, 0.88), major osteoporotic fracture (HR: 0.66; 95% CI: 0.45, 0.95), and hip fracture (HR: 0.58; 95% CI: 0.36, 0.95).
Dostal, 2016 [147]	Model: a randomized, double-blind, placebo-controlled clinical trial. This substudy was conducted in 121 overweight/obese participants [body mass index (BMI) (kg/m^2^) ≥ 25.0]. Treatments: placebo-controlled clinical trial in 937 postmenopausal women (aged 50–70 y) assigned to receive either GTE containing 843 mg (−)-epigallocatechin-3-gallate or placebo	Compared to control group, GTE groups: ↔ BMI, total fat mass), percentage of body fat, or BMD ↔ circulating leptin, ghrelin, adiponectin, or insulin concentrations ↓ tissue %fat during the intervention as baseline BMI increased
Huang, 2018 [139]	Model: Cross-sectional study Treatments: A total of 1495 Chinese women aged more than 40 years were included. Tea consumption, socio-demographic information, and lifestyle habits were collected by a face-to-face questionnaire.	Compared to non-consumers, tea consumption group: ↑ approximately 1.9% higher BMD ↓ 3.6% lower BR
Shen, 2018 [140]	Model: A Prospective Cohort Study Treatments: 453,625 participants from the China Kadoorie Biobank (CKB). Tea consumption was self-reported at baseline. Hospitalized fractures were ascertained through linkage with local health insurance claim databases.	Compared to non-consumers, tea consumption group: ↓ risk of any fracture in daily tea consumption ↓ risk of hip fracture in those who had drunk tea for more than 30 years
Li, 2019 [141]	Model: a population-based study Treatments: 20,643 participants from the China Kadoorie Biobank (CKB), who have finished both baseline survey (2004–2008) and a re-survey (2013–2014). They were aged 38–86 y at re-survey. Tea consumption was self-reported at both baseline and re-survey.	Compared to non-consumers, prolonged weekly tea consumers: ↑ calcaneus BMD ↔ BMD measures with the amount of tea leaves added ↔ Tea consumption was not associated with calcaneus BMD measures in men

ALP, alkaline phosphatase; BMD, Bone mineral density; BMP-2, Bone Morphogenetic Protein 2; BR, buckling ratio; COX, cyclooxygenase; EGCG, epigallocatechin gallate; FHP, free hydroxyproline; GAG, glycosaminoglycan; GT, green tea; GTE, green tea extract; GTP, green tea polyphenols; iNOS, inducible nitric oxide synthase; IL-1β, interleukin-1β; LPS, lipopolysaccharide; MREI, molar roots exposure index; NANA, N-acetylneuraminic acid; NF-κB, nuclear factor kappa-B; NO, nitric oxide; RANKL, Receptor activator of nuclear factor kappa-Β ligand; RUNX2, Runt-related transcription factor 2; OPG, osteoprotegerin; OVX, ovariectomized; SG, salivary glands; TC, Tai Chi; TNF-α, tumor necrosis factor-α; ↑, increase; ↓, decrease; ↔, no change.
